# Large Language Model–Based Assessment of Clinical Reasoning Documentation in the Electronic Health Record Across Two Institutions: Development and Validation Study

**DOI:** 10.2196/67967

**Published:** 2025-03-21

**Authors:** Verity Schaye, David DiTullio, Benedict Vincent Guzman, Scott Vennemeyer, Hanniel Shih, Ilan Reinstein, Danielle E Weber, Abbie Goodman, Danny T Y Wu, Daniel J Sartori, Sally A Santen, Larry Gruppen, Yindalon Aphinyanaphongs, Jesse Burk-Rafel

**Affiliations:** 1 Department of Medicine NYU Grossman School of Medicine New York, NY United States; 2 Institute for Innovations in Medical Education NYU Grossman School of Medicine New York, NY United States; 3 Division of Applied AI Technologies NYU Langone Health New York, NY United States; 4 Department of Biostatistics, Health informatics, and Data Sciences University of Cincinnati College of Medicine Cincinnati, OH United States; 5 Division of Hospital Medicine, Department of Pediatrics, Cincinnati Children's Hospital Medical Center University of Cincinnati College of Medicine Cincinnati, OH United States; 6 Division of Hospital Medicine, Department of Internal Medicine University of Cincinnati College of Medicine Cincinnati, OH United States; 7 Department of Emergency Medicine University of Cincinnati College of Medicine Cincinnati, OH United States; 8 Department of Learning Health Sciences University of Michigan Medical School Ann Arbor, MI United States

**Keywords:** large language models, artificial intelligence, clinical reasoning, documentation, assessment, feedback, electronic health record

## Abstract

**Background:**

Clinical reasoning (CR) is an essential skill; yet, physicians often receive limited feedback. Artificial intelligence holds promise to fill this gap.

**Objective:**

We report the development of named entity recognition (NER), logic-based and large language model (LLM)–based assessments of CR documentation in the electronic health record across 2 institutions (New York University Grossman School of Medicine [NYU] and University of Cincinnati College of Medicine [UC]).

**Methods:**

The note corpus consisted of internal medicine resident admission notes (retrospective set: July 2020-December 2021, n=700 NYU and 450 UC notes and prospective validation set: July 2023-December 2023, n=155 NYU and 92 UC notes). Clinicians rated CR documentation quality in each note using a previously validated tool (Revised-IDEA), on 3-point scales across 2 domains: differential diagnosis (D0, D1, and D2) and explanation of reasoning, (EA0, EA1, and EA2). At NYU, the retrospective set was annotated for NER for 5 entities (diagnosis, diagnostic category, prioritization of diagnosis language, data, and linkage terms). Models were developed using different artificial intelligence approaches, including NER, logic-based model: a large word vector model (scispaCy en_core_sci_lg) with model weights adjusted with backpropagation from annotations, developed at NYU with external validation at UC, NYUTron LLM: an NYU internal 110 million parameter LLM pretrained on 7.25 million clinical notes, only validated at NYU, and GatorTron LLM: an open source 345 million parameter LLM pretrained on 82 billion words of clinical text, fined tuned on NYU retrospective sets, then externally validated and further fine-tuned at UC. Model performance was assessed in the prospective sets with *F*_1_-scores for the NER, logic-based model and area under the receiver operating characteristic curve (AUROC) and area under the precision-recall curve (AUPRC) for the LLMs.

**Results:**

At NYU, the NYUTron LLM performed best: the D0 and D2 models had AUROC/AUPRC 0.87/0.79 and 0.89/0.86, respectively. The D1, EA0, and EA1 models had insufficient performance for implementation (AUROC range 0.57-0.80, AUPRC range 0.33-0.63). For the D1 classification, the approach pivoted to a stepwise approach taking advantage of the more performant D0 and D2 models. For the EA model, the approach pivoted to a binary EA2 model (ie, EA2 vs not EA2) with excellent performance, AUROC/AUPRC 0.85/ 0.80. At UC, the NER, D-logic–based model was the best performing D model (*F*_1_-scores 0.80, 0.74, and 0.80 for D0, D1, D2, respectively. The GatorTron LLM performed best for EA2 scores AUROC/AUPRC 0.75/ 0.69.

**Conclusions:**

This is the first multi-institutional study to apply LLMs for assessing CR documentation in the electronic health record. Such tools can enhance feedback on CR. Lessons learned by implementing these models at distinct institutions support the generalizability of this approach.

## Introduction

Clinical reasoning (CR) is a fundamental skill that requires incorporating vast amounts of information into a prioritized differential diagnosis and treatment plan and therefore crucial that trainees are given feedback to improve [1]. Documentation in the electronic health record (EHR) can provide this opportunity. Furthermore, poor documentation can reflect lack of refined CR and has been hypothesized to be linked to diagnostic errors [2-4]. There are established human ratings tools to provide feedback on CR documentation such as the Revised-IDEA tool, a rubric that facilitates giving feedback in 4 essential domains of CR, including interpretive summary, differential diagnosis, explanation of lead diagnosis, and explanation of alternative diagnoses [5]. However, feedback provided to trainees can still be limited due to faculty having different standards of what constitutes high quality CR documentation and limited time for feedback in the fast-paced clinical environment [5-7].

Machine learning (ML), natural language processing (NLP), and other artificial intelligence (AI) technologies have emerged as avenues to augment feedback [8-12]. NLP has been used to automate the scoring of documentation in simulated scenarios [13-16]. AI-augmented assessment of CR documentation has also been implemented in clinical environments; we have previously reported on an NLP-based supervised ML model that provides feedback on internal medicine (IM) residents CR documentation [17]. However, this model was developed using earlier technologies and only provides binary feedback. Similarly, Feldman et al [18] published the development of a supervised ML model that provides binary feedback on CR documentation (quality of prioritized differential in progress notes).

The more recent advances of generative AI (GAI) and large language models (LLMs) have expanded the potential of AI as a powerful tool to augment feedback [19,20]. There is a building body of literature on the use of LLMs in CR tasks demonstrating that AI outperforms humans [21-25]. Among these studies is a recent article by Goh et al [21] concluding that LLMs outperformed humans and humans plus LLMs on clinical vignettes as assessed by a standardized rubric of diagnostic performance. In terms of use of LLMs for assessment and feedback on CR, Çiçek et al [26] published on the use of ChatGPT versus expert written feedback on CR questions showing mixed reception from the medical students receiving the feedback. Finally, Jamieson et al [27] used AI to provide feedback on medical student Objective Structured Clinical Examination postencounter notes, an important method of assessing CR, and showed high agreement between AI and expert human ratings. However, these studies are all conducted with curated medical data, at single institutions, and do not focus on assessment of human reasoning but on performance of LLM reasoning as compared with humans.

Navigating the use of LLMs with EHR data (vs curated data) can be much more complicated. There are the challenges of accessing the right data from the chart, a higher burden of accuracy, privacy issues, and variability in EHRs [28-30]. In addition, initial studies have shown LLMs do not perform as well digesting the complexity of information in the EHR to make accurate diagnoses [30]. Finally, while the performance of LLMs on CR tasks is promising, it is far from sufficient to replace humans and it is essential that we continue to provide feedback on our learners’ CR [19,30,31]. AI-based tools remain an important strategy to enhance the amount of feedback we provide [12].

Here, we report on an expansion of our previous work and describe the development across 2 institutions of a named entity recognition (NER), logic-based assessment and LLM-based assessments of IM resident CR documentation in the EHR that predicts the quality of CR across 2 domains using the Revised-IDEA tool.

## Methods

### Setting and Study Population

We conducted this study at 2 institutions, New York University Grossman School of Medicine (NYU) and University of Cincinnati College of Medicine (UC). NYU is a northeastern academic medical center with multiple hospital sites; the NYU IM residency program has 2 resident populations with separate recruitment processes and clinical rotations at different hospitals that use the same EHR, NYU Langone Health Manhattan and NYU Langone Health Brooklyn. NYU residents also rotate at 2 other sites with distinct EHRs not included in this study. In terms of technical resources, NYU has the infrastructure of both the Institute for Innovations in Medical Education which is a multidisciplinary team of clinician educators, data scientists, informaticians, and developers who apply the science of education and informatics to transform teaching, learning, evaluation, and assessment at NYU and the Division of Applied AI Technologies which focuses on using data and modeling to predict health outcomes across NYU Langone Health [32,33]. In addition, both education and EHR data are stored and easily accessible through a central education data warehouse and there is access to a distributed-memory, high-performing computing cluster [34,35].

UC is a midwestern academic medical center within which IM residents rotate at University of Cincinnati Medical Center (UCMC) and the Veterans Affairs Medical Center; only notes written at UCMC were included in the study. In terms of technical resources, UC has the Department of Biostatistics, Health Informatics, and Data Sciences (BHIDS) that enables the UC academic health care enterprise to make better use of biomedical data and technology for new discoveries, innovative science, and improved health care [36]. Although UC has access to many data sources across the health system, medical school and other education programs, currently there is not a centralized database for education and EHR data like at NYU. In addition, UC has some access to high-performing computing resources, but NYU has a more developed infrastructure for using these tools for both clinical and educational use than at UC.

**Figure 1:**
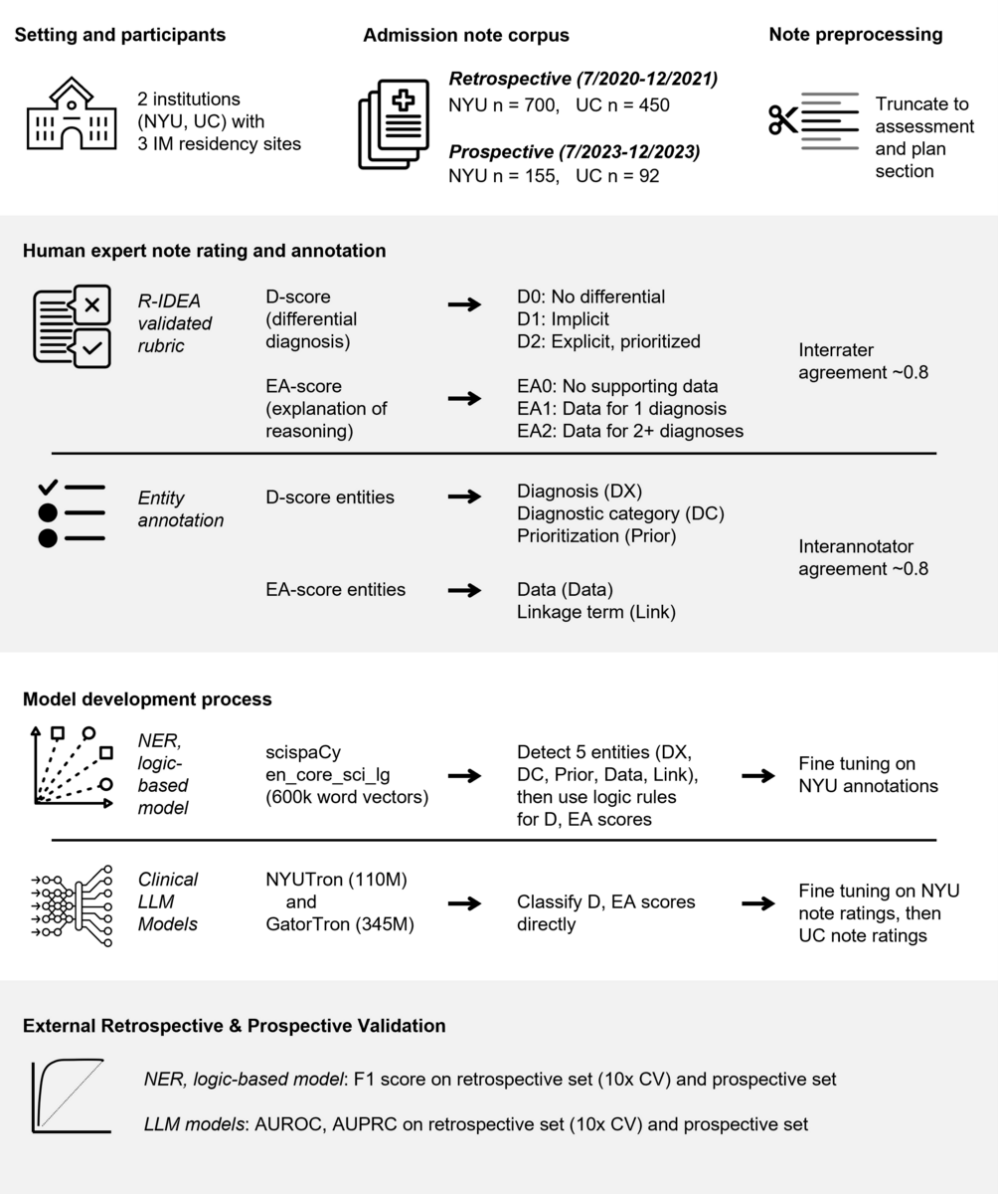
Overview of the development and validation across 2 institutions of named entity recognition: logic-based assessment and large language model–based assessments of resident clinical reasoning documentation in the electronic health record. AUROC: area under the precision-recall curve; AURPC: area under the precision-recall curve; CV: cross-validation; LLM: large language model; NER: named entity recognition; NYU: New York University Grossman School of Medicine; UC: University of Cincinnati College of Medicine.

At each site 2 note sets were retrieved from an integrated EHR (Epic Systems): (1) retrospective dataset comprised of IM resident admission notes from July 2020-December 2021 (n=700 NYU notes, n=450 UC notes) and (2) prospective validation dataset from July 2023-December 2023 (n=155 NYU notes, n=92 UC notes; Figure 1). These time periods were selected to ensure a sufficient range of diagnoses and residents were represented and that COVID-19 admissions were not overrepresented. The datasets at NYU were larger because the initial plan was for primary development and model fine-tuning occurring at this institution.

### Human Note Rating

We used the DEA components of the Revised-IDEA tool as our human rating gold standard [5]. We maintained the original D (differential diagnosis) score whether a note has an explicitly prioritized differential diagnosis with specific diagnoses (eg, not diagnostic categories such as cardiac), scored as D0, D1, or D2. We discovered early in training experiments that discerning the E (explanation of lead diagnosis) and A (alternative diagnosis explained) would be difficult with available AI models, and iterated to create an overall explanation of reasoning EA score combining the E and A components (ie, EA0, EA1, or EA2; Figure 1; Multimedia Appendix 1). In total, 6 faculty (clinician educators with expertise in CR, assessment, and psychometrics), 1 resident, and 1 medical student reviewed admission notes to create the new EA score.

On the NYU retrospective dataset, raters also annotated spans of text using Prodigy (an annotation tool for creating training data for ML models). Annotations included 5 entity types for NER, 3 components of the D score (diagnosis [Dx], diagnostic category [DC], prioritization of diagnosis language [Prior]) and 2 components of the EA score (data [Data] and linkage terms [Link]).

To demonstrate interrater reliability, 2 raters labeled 76 notes from the NYU retrospective set for NER, D, and EA scores. For the UC notes, 1 rater from NYU and 2 raters from UC rated 20 notes for D and EA scores. Intraclass correlation (ICC) using a 2-way ANOVA with mixed effects was calculated to assess rater consistency. The remainder of the notes at each institution were rated by 1 rater for D and EA scores. The remainder of the retrospective set at NYU was also rated by 1 rater for NER.

### Note Preprocessing

We aimed to isolate the section of the assessment that concentrates on the differential diagnosis and the explanation of reasoning for the primary presenting problem. We iterated on previous truncation strategies outlined previously by Schaye et al [17] and different approaches were required at each institution given different note writing styles (details in Multimedia Appendix 2).

### Model Development

Model training occurred with several approaches, NYU development of NER, logic-based model with subsequent external validation at UC, NYU fine tune training of LLM NYUTron [37], NYU fine-tune training of LLM GatorTron [38], UC external validation and fine tune training of NYU fine-tuned GatorTron, and UC fine tune training of GatorTron. We were not able to validate NYUTron at UC pending contract execution for data sharing and will ideally do so in the future.

The selection of these model architectures was driven by their proven effectiveness in handling clinical text data and their ability to capture complex semantic relationships [37-39]. The NER, logic-based model was chosen for its capability to leverage domain-specific knowledge and predefined rules, which is particularly useful for structured data extraction in clinical settings. On the other hand, the LLMs were selected for their advanced contextual understanding and scalability, making them suitable for more nuanced classification tasks.

#### NER, Logic-Based Model Approach

We used a large, NLP word embedding model trained on scientific texts from the scispaCy library (en_core_sci_lg) with more than 700k vocabulary and 600k word vectors [39]. Word embeddings are a type of word representation that allows words to be represented as vectors in a continuous vector space, capturing semantic meanings. We adjusted model weights with backpropagation (a method used to minimize the error in predictions by adjusting the weights of the model) using the human-annotated labels of the 5 entity types (Dx, DC, Prior, Data, and Link).

We calculated the predicted D scores (ie, D0, D1, and D2) using logic-based relationships between the extracted entities and the rating scale: D0, fewer than 2 unique diagnoses (Dx entity counts); D2, 2 unique diagnoses (Dx entity counts); and explicit prioritization (Prior entity counts); D1, everything else. We attempted ML models to predict EA score from the named entities; however, due to poor model performance, we abandoned further attempts to develop NER, logic-based EA scores.

In order to provide an impartial and dependable evaluation of the model’s performance, we used 10-fold cross-validation (a technique where the data is divided into ten parts, and the model is trained and validated 10 times, each time using a different part as the validation set and the remaining parts as the training set). We calculated the Type NER (which demands some overlap between the system tagged entity and the gold standard annotation) evaluation metric, as suggested in SemEval-2013 Task 9, at each k-fold (details in Multimedia Appendix 3) [40]. We report *F*_1_-score over 10-fold runs for each D score entity type (Dx, DC, and Prior) and D score prediction. The *F*_1_-score is crucial for evaluating NER-based models because it balances precision and recall (precision measures the accuracy of the positive predictions, while recall measures the ability to find all relevant instances), providing a comprehensive measure of a model’s ability to correctly identify entities while minimizing both false positives and false negatives. This balance is essential for handling the often-imbalanced nature of entity distributions in text, ensuring a more accurate assessment of model performance. We shared the best performing NER, logic-based D model with UC through a docker container and externally validated the model on the UC retrospective set.

#### LLM Approaches

NYUTron, developed by NYU, is a BERT (Bidirectional Encoder Representation with Transformer)-like LLM with about 110 million parameters that has been pretrained on 7.25 million clinical notes (4.1 billion words, notes through May 2020) [37]. We fine-tuned the model to classify D and EA scores. We applied a 1-versus-rest approach, which resulted in the development and testing of 6 distinct models, each corresponding to a different D and EA score category (ie, D0, D1, D2, EA0, EA1, and EA2 models). However, EA0 and EA1 models did not have adequate performance so we pivoted our approach to create a single binary EA2 model (ie, EA2 vs not EA2). To evaluate model performance, we used 10-fold cross-validation, with area under the receiver operating characteristic curve (AUROC) and area under the precision-recall curve (AUPRC) averaged over the 10 runs. We chose AUROC and AUPRC as our primary metrics for all LLM-based models because these metrics are favored over *F*-scores for binary classification tasks, particularly with imbalanced datasets. They assess model performance across all possible thresholds, offering a more detailed understanding of trade-offs between true positives, false positives, and precision-recall dynamics, thereby aiding in the identification of the optimal decision-making threshold, which a single *F*-score cannot provide.

Unlike NYUTron, GatorTron is an open source LLM with 345 million parameters that was pretrained on over 82 billion words of deidentified clinical text [38]. To enhance generalizability using an open source LLM, the same experiments described above for NYUTron were taken with GatorTron at NYU.

The NYU fine-tuned GatorTron EA2 model was shared with UC, which conducted external validation, and further fine-tuned following a similar process. Due to the smaller set of notes and hardware limitations, particularly a relatively small Video Random Access Memory size of 16GB, some modifications were applied. A runtime text augmentation was implemented during training with the following settings: 15 words of synonym replacement, random word insertion, and random swap each, and finally, a random word deletion of 15% probability. Finally, we applied random minority oversampling using inverse class frequency during training.[41] In addition, given these limitations, we did not attempt to fine-tune the 3 separate NYU fine-tuned GatorTron D models at UC. Instead, we fine-tuned the original GatorTron model to predict all 3 possible D Scores with a single model at UC using the same training process and hyperparameters as the EA2 model. Further details on model hyperparameters and packages used at NYU and UC can be found in Multimedia Appendix 4.

#### Prospective Validation

As a final step of validation, we ran each of the best performing models selected for implementation on the site’s prospective validation sets and assessed performance using *F*_1_-score for the NER, logic-based model and AUROC and AUPRC for the LLM models.

### Ethical Considerations

The study was approved by the NYU and UC institutional review boards, (i19-00280) and (2022-058), respectively. A waiver of consent was obtained for retrospective chart reviews. All retrospective data were anonymized when possible and appropriate measures taken to protect participant information. We followed the reporting guidelines by Klement and El Emam [42] for prognostic and diagnostic machine learning studies.

## Results

### Human Note Rating

**Figure 2:**
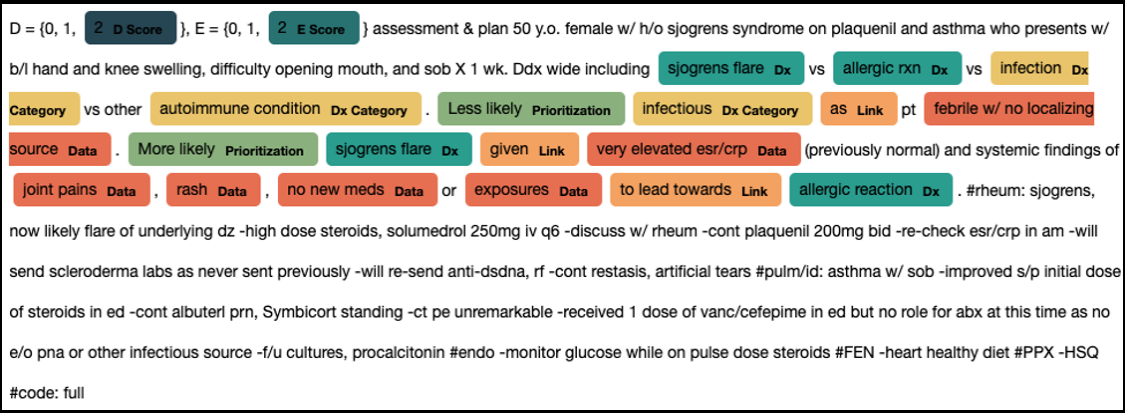
Example note (modified to protect patient privacy) with human rating of D and EA scores and annotation for named entity recognition of 5 entity types: 3 components of the D score (diagnosis [Dx], diagnostic category (DC], and prioritization of diagnosis language [Prior]) and 2 components of the EA score (data [Data] and linkage terms [Link]).

At NYU, ICC was 0.83 (95% CI 0.74-0.89) and 0.77 (95% CI 0.65-0.85) for the D and EA scores, respectively, indicating substantial interrater agreement. Interannotator agreement across all 5 entity types averaged *F*_1_-score=0.81 (range 0.71-0.87 by entity type), indicating strong annotator overlap (Figure 2). At UC, ICC was 0.83 (95% CI 0.68-0.92) and 0.84 (95% CI 0.70-0.93) for the D and EA scores, respectively.

In both datasets, at each institution there was a range of human-rated D and EA scores, diagnoses, and patient demographics mitigating concerns of potential bias in the selection of datasets (Table 1).

**Table 1:** Descriptive statistics of human-rated note quality using the Revised-DEA tool and patient characteristics in the retrospective and prospective note sets at NYU^a^ and UC^b^.

Characteristics		NYU retrospective note set (n=700)	NYU prospective note set (n=155)	UC retrospective note set (n=450)	UC prospective note set (n=92)
**D score n (%)**					
	0	109 (15.6)	55 (35.5)	120 (26.7)	17 (18.5)
	1	154 (22)	46 (29.7)	155 (34.4)	31 (33.7)
	2	437 (62.4)	54 (34.8)	175 (38.9)	44 (47.8)
**E score n (%)**					
	0	73 (10.4)	19 (12.3)	96 (21.3)	27 (29.3)
	1	255 (36.4)	74 (47.7)	171 (38)	21 (22.8)
	2	372 (53.1)	62 (40)	183 (40.7)	43 (46.7)
**Patient age (years), n (%)**					
	≤ 54	142 (20.3)	29 (18.7)	147 (32.7)	25 (27.2)
	55-68	181 (25.9)	35 (22.6)	184 (40.9)	37 (40.2)
	69-80	236 (33.7)	45 (29)	86 (19.1)	20 (21.7)
	≥ 81	141 (20.1)	33 (21.3)	33 (7.3)	10 (10.9)
	Not answered	0 (0)	13 (8.4)	0 (0)	0 (0)
**Patient sex, n (%)**					
	Female	340 (48.6)	67 (43.2)	215 (47.8)	52 (56.5)
	Male	360 (51.4)	75 (48.4)	234 (52)	40 (43.5)
	Not answered	0 (0)	13 (8.4)	1 (0.2)	0 (0)
**Primary diagnosis by ICD-10^c^, n (%)**					
	Cardiac	134 (19.1)	38 (24.5)	42 (8.8)	12 (12.6)
	Dermatologic	13 (1.9)	3 (1.9)	18 (3.8)	4 (4.2)
	Endocrine	26 (3.7)	7 (4.5)	27 (5.7)	3 (3.2)
	Gastrointestinal	68 (9.7)	15 (9.7)	56 (11.8)	8 (8.4)
	Genitourinary	49 (7)	14 (9)	39 (8.2)	10 (10.5)
	Hematologic or oncologic	61 (8.7)	7 (4.5)	35 (7.4)	5 (5.3)
	Infectious	117 (16.7)	21 (13.5)	10 (2.1)	2 (2.1)
	Musculoskeletal	19 (2.7)	3 (1.9)	17 (3.6)	7 (7.4)
	Neurologic	21 (3.)	1 (0.6)	12 (2.5)	4 (4.2)
	Other	54 (7.7)	11 (7.1)	149 (31.4)	30 (31.6)
	Psychiatric	5 (0.7)	7 (4.5)	13 (2.7)	2 (2.1)
	Pulmonary	72 (10.3)	10 (6.5)	47 (9.9)	7 (7.4)
	Not answered	61 (8.7)	18 (11.6)	10 (2.1)	1 (1.1)

^a^NYU: New York University Grossman School of Medicine.

^b^UC: University of Cincinnati College of Medicine.

^c^ *ICD-10: International Statistical Classification of Diseases, Tenth Revision.*

### Model Performance

#### NER Logic-Based Model

In the NYU retrospective dataset, the NER *F*_1_-score for the entity types used to compute the D score (Dx, DC, and Prior) was 0.66. The NER model performed the best in extracting Prior and Dx entities with an *F*_1_-score of 0.75 and 0.68, respectively, but struggled with DC entities, achieving a 0.37 *F*_1_-score.

The NER, D logic-based model performed well at both sites with *F*_1_-scores of 0.83, 0.78, and 0.75 for D0, D1, and D2 scores, respectively at NYU and *F*_1_-scores of 0.75, 0.71, 0.76 for D0, D1, D2 scores, respectively at UC. At UC, the NER, D-logic–based model was the best performing D model overall selected for implementation and run on the UC prospective validation set with *F*_1_-scores of 0.80, 0.74, and 0.80 for D0, D1, D2 scores, respectively.

#### LLM-Based Models

**Figure 3:**
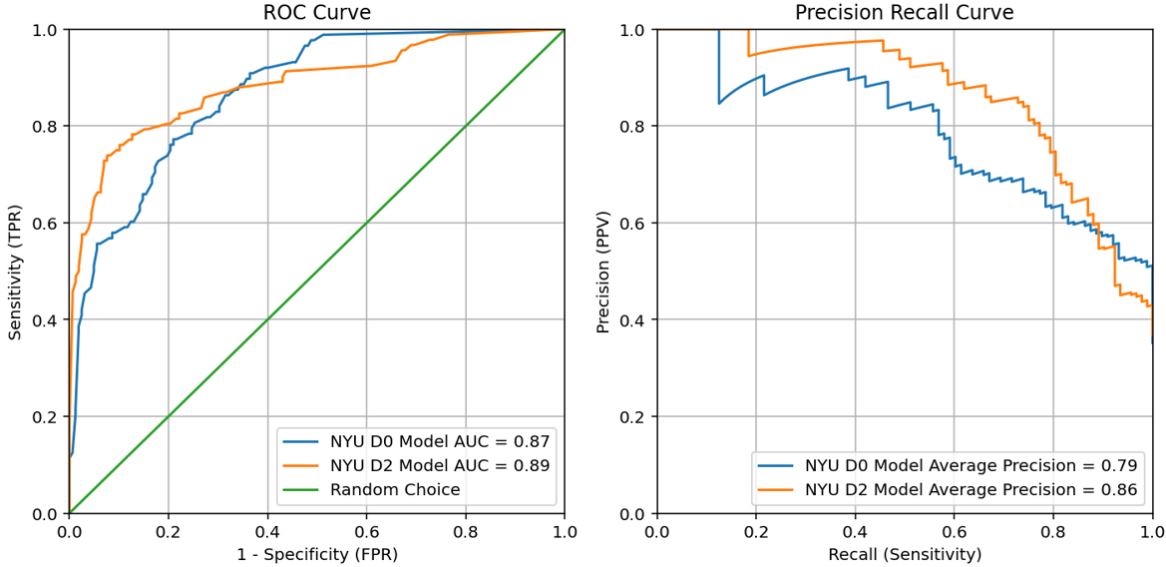
Large language model performance on prospective note sets classifying differential diagnosis (D0 and D2) in resident admission notes for best performing D models selected for implementation at New York University Grossman School of Medicine. AUC: area under the curve; FPR: false positive rate; NYU: New York University; PPV: positive predictive value; ROC: receiver operating characteristic; TPR: true positive rate.

At NYU, NYUTron overall had better D and EA model performance on the retrospective set than GatorTron and were the best performing models overall (Table 2). However, while the D0 and D2 NYUTron models performed well, the D1 model was not performant on the retrospective set (AUROC 0.57, 95% CI 0.53-0.69; AUPRC 0.33, 95% CI 0.26-0.43) and therefore was not suitable for implementation. As such, a stepwise approach was taken for the D1 model by taking advantage of the more performant D0 and D2 models (Table 2). The D0 and D2 NYUTron models had excellent performance on the prospective dataset as follows: D0 model, AUROC 0.87 and AUPRC 0.79 and D2 model, AUROC 0.89 and AUPRC 0.86 (Figure 3).

**Figure 4:**
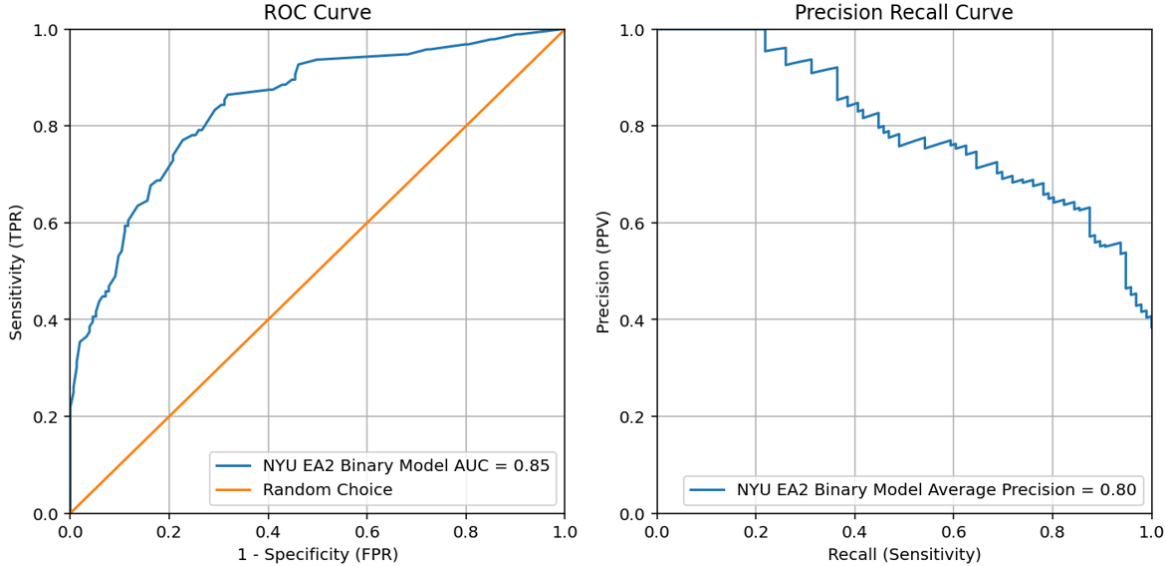
Large language model performance on prospective note sets classifying explanation of reasoning (EA2) in resident admission notes for best performing EA models selected for implementation at New York University Grossman School of Medicine. AUC: area under the curve; FPR: false positive rate; NYU: New York University; PPV: positive predictive value; ROC: receiver operating characteristic; TPR: true positive rate.

Both the NYUTron EA0 and EA1 models had insufficient performance for implementation therefore the approach pivoted to create a single binary EA model, EA2 vs not EA2 (ie, EA0 or EA1; Table 2). The binary NYUTron EA2 model achieved sufficient performance for implementation with an AUROC 0.85 and AUPRC 0.80 on the prospective dataset (Figure 4).

**Figure 5:**
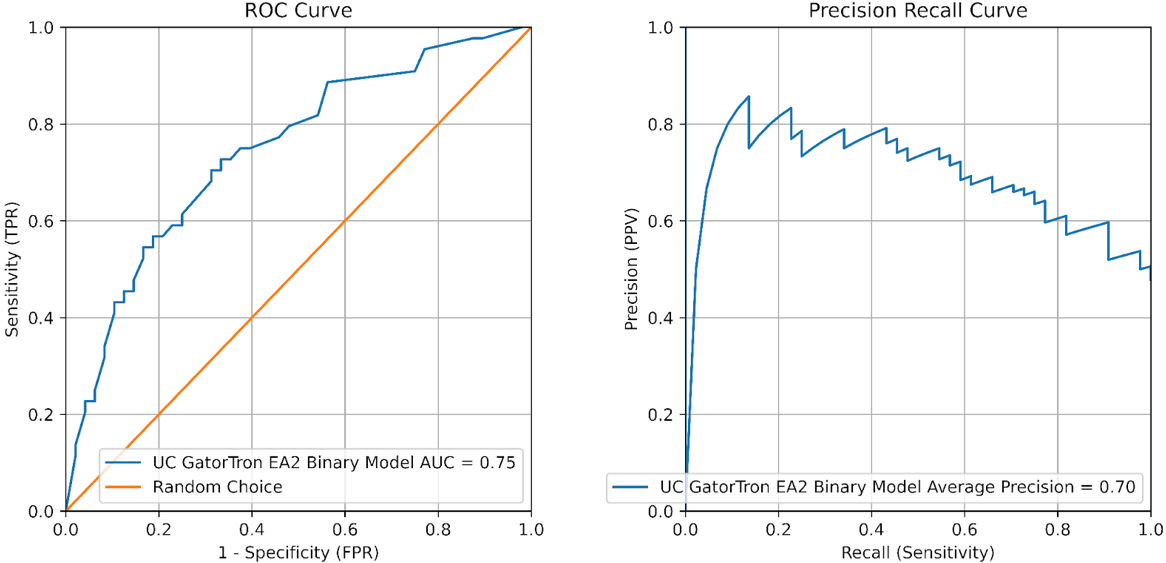
Large language model performance on prospective note sets classifying explanation of reasoning (EA2) in resident admission notes for best performing EA models selected for implementation at University of Cincinnati College of Medicine. AUC: area under the curve; FPR: false positive rate; PPV: positive predictive value; ROC: receiver operating characteristic; TPR: true positive rate; UC: University of Cincinnati.

In external validation at UC, the NER, D logic model performed better than the D GatorTron models and were the D models implemented as described above (Table 2). The GatorTron EA2 model did reach sufficient performance for subsequent prospective validation with an AUROC 0.75 and AUPRC 0.69 (Table 2 and Figure 5).

A final step in optimizing performance was selecting thresholds for all LLM models implemented (details in Multimedia Appendix 5).

**Table 2:** Large Language Model (LLM) performance on retrospective note sets for all NYUTron and GatorTron experiments classifying differential diagnosis and explanation of reasoning scores in resident admission notes at NYU^a^ and UC^b^.

LLM^c^	Site	D/EA score classification	AUROC^d^ retrospective validation, (95% CI)	AUPRC^e^ retrospective validation, (95% CI)
NYUTron	NYU	D0	0.91 (0.85-0.93)	0.72 (0.58-0.76)
NYUTron	NYU	D1	0.57 (0.53-0.69)	0.33 (0.26-0.43)
NYUTron	NYU	D2	0.81 (0.8-0.87)	0.89 (0.85-0.93)
NYUTron	NYU	D1 stepwise approach^f^	—^g^	—
NYUTron	NYU	EA0	0.83 (0.72-0.86)	0.36 (0.23-0.47)
NYUTron	NYU	EA1	0.74 (0.67-0.78)	0.63 (0.54-0.68)
NYUTron	NYU	EA2	0.84 (0.8-0.87)	0.84 (0.81-0.89)
NYUTron	NYU	EA2 binary model^h^	0.84 (0.81-0.85)	0.82 (0.8-0.87)
GatorTron	NYU	D0	0.92 (0.84-0.94)	0.72 (0.48-0.75)
GatorTron	NYU	D1	0.54 (0.5-0.59)	0.31(0.24-0.37)
GatorTron	NYU	D2	0.73 (0.78-0.85)	0.80 (0.82-0.92)
GatorTron	NYU	D1 stepwise approach^f^	—	—
GatorTron	UC	D0	0.75 (0.54-0.96)	0.51 (0.23-0.79)
GatorTron	UC	D1	0.61 (0.44-0.78)	0.46 (0.22-0.7)
GatorTron	UC	D2	0.72 (0.61-0.83)	0.63 (0.46-0.79)
GatorTron	NYU	EA0	0.80 (0.73-0.89)	0.42 (0.25-0.53)
GatorTron	NYU	EA1	0.75 (0.62-0.78)	0.63 (0.48-0.67)
GatorTron	NYU	EA2	0.83 (0.76-0.87)	0.83 (0.79-0.9)
GatorTron	NYU	EA2 binary model^h^	0.81 (0.76-0.87)	0.80 (0.79-0.9)
GatorTron	UC	EA0	—	—
GatorTron	UC	EA1	—	—
GatorTron	UC	EA2	—	—
GatorTron	UC	EA2 binary model^h^	0.72 (0.51-0.93)	0.63 (0.41-0.85)

^a^NYU: New York University Grossman School of Medicine.

^b^UC: University of Cincinnati College of Medicine.

^c^LLM: large language model.

^d^AUROC: area under the receiver operating characteristic curve.

^e^AUPRC: area under the precision-recall curve.

^f^The D1 model ultimately did not have sufficient performance for implementation while the D0 and D2 had excellent performance so a stepwise approach was taken for the D1 score: (1) If D0 model predicts D=0, then D=0, (2) If D2 model predicts D=2, then D=2, and (3) Else D=1. The NYU D1 Stepwise Approach achieved precision of 0.79 while the GatorTron D1 Stepwise Approach achieved precision of 0.73.

^g^Not applicable.

^h^Both the EA0 and EA1 models had insufficient performance for implementation therefore the approach pivoted to create a single EA model EA2 vs not EA2.

## Discussion

### Principal Findings

We developed both NER, logic-based and LLM-based assessments of CR documentation in the EHR across 2 institutions with different residency training cultures, expectations for documentation, and technical resources. This builds upon previous work on a supervised ML model of Schaye et al [17] to assess CR documentation, generating more specific feedback across 2 domains of the Revised-IDEA tool. We developed high performing D models that can provide feedback on a 3-point scale and an EA model that can provide feedback on a 2-point scale with comparable performance with our earlier model that provides only binary feedback. Compared with our previous model, which used logistic regression and cTAKES for binary classification of CR documentation quality, our current models leverage advanced NLP techniques, including LLMs, and provides more granular feedback. In addition, the current models were designed at 1 institution and then externally validated at a second institution, enhancing their generalizability and robustness. To our knowledge this is the first study to apply LLMs to human CR in EHR data across institutions (rather than LLM reasoning or human reasoning on curated medical data) [17,18,21-24,26,27,30]. Furthermore, despite the advances in LLMs, these technologies are not yet performant to replace human reasoning. AI-based tools such as the ones we developed can help ensure we are continuing to give our trainees feedback on the essential human skill of CR [12,19,30,31].

While we were able to navigate successfully some of the challenges of working with LLMs and EHR data such as accessing the right data from the chart and privacy issues [28-30], we were not able to achieve sufficient performance of all the models at both sites. The performance differences between NYUTron, GatorTron, and the NER, logic-based model can be attributed to several factors. NYUTron, developed specifically on NYU EHR data, likely benefited from being fine-tuned on a dataset closely aligned with its pretraining data, which may have contributed to its superior performance at NYU. This alignment could have allowed NYUTron to better capture the nuances and specificities of the documentation style and clinical language used at NYU. In contrast, GatorTron, while being a robust open-source LLM with a larger parameter set, may not have been as finely attuned to the specific documentation styles at NYU or UC, leading to relatively lower performance. The NER, logic-based model, leveraging domain-specific knowledge and predefined rules, demonstrated strong performance in structured data extraction tasks, particularly at UC, where it outperformed the GatorTron models for D score predictions. This suggests that for certain structured tasks, smaller, more focused NLP models can sometimes be more effective than larger, more generalized LLMs. In addition, the annotation processes and the quality of the training data, including the consistency and accuracy of human annotations, likely played a significant role in influencing model performance.

It might not always be the newest technology needed to solve the task at hand and comparison of performance of different technologies can be a helpful strategy. Of note when this work initially began, GAI models such as ChatGPT were not readily available in HIPAA (Health Insurance Portability and Accountability Act)-compliant instances at either institution but will be technology we integrate into future work.

In terms of the performance of the models we plan to implement, the significance of the *F*_1_-scores and AUROC metrics is crucial for understanding their practical implications in a clinical environment. Of the NER, logic-based models we plan to implement at UC only the D1 model has an *F*_1_-score less than 0.80. An *F*_1_-score of greater than 0.80 indicates a high level of accuracy in identifying relevant entities, which translates to reliable feedback being generated by the D0 and D2 NER, logic-based models each with *F*_1_-scores of 0.80. While the D1 NER, logic-based model did not reach this level of performance with an *F*_1_-score of 0.74, an *F*_1_-score greater than 0.70 has been deemed sufficient for applications not involving direct patient care decisions, such as for documentation feedback and educational purposes [43,44]. Furthermore, in the context of educational feedback, an *F*_1_-score of greater than 0.70 ensures a balance between precision and recall, which is crucial for providing comprehensive and reliable feedback to residents without the risk of significant negative consequences [45]. Similarly, all of the LLMs we plan to implement at NYU and UC except 1 have AUROCs greater than 0.80; the GatorTron LLM EA2 model being the exception with an AUROC 0.75. An AUROC of greater than 0.80 suggests that the model has an excellent ability to accurately perform a classification task in this case classify quality of CR documentation. Similar to *F*_1_-scores, an AUROC greater than 0.70 is acceptable in particular for low stakes clinical tasks like formative feedback on CR documentation being provided with these models [46,47].

Finally, we also learned a lot of lessons working across two institutions on how AI technologies can be adapted and successfully implemented at sites with different resources. Some key takeaways include experimenting with different LLMs including ones that are openly available, performing primary development at an institution with more resources and creating a HIPAA-compliant pipeline to share code thus mitigating ethical concerns of patient privacy, developing variations on truncation methods to account for different note writing styles, and creating adaptable approaches to different degrees of computing power such as using text augmentation to prevent overfitting at UC. We will take these lessons learned about generalizability to the next phases of the work and develop strategies to implement more advanced technologies across institutions while maintaining HIPAA compliance working with EHR data.

Our next steps include integrating these AI-based assessments into each residency program as new mechanisms to provide formative feedback to residents. At NYU, we will iterate on dashboards implemented with our previous supervised ML model to improve resident and faculty coaches’ ability to visualize longitudinal trends and set improvement goals [17]. At UC, dashboard implementation will represent a new mechanism for formative feedback for residents that currently does not exist. Dashboards at both programs will include percent high-quality (score of 2) notes for D score and E score, trends in scores over time, and goal targets for residents. Residents at both programs will be able to access their data through dashboards for routine review. Each program will create a process for residents to review their performance data and set specific, individualized goals for improving their CR documentation. After initial implementation, we will collect data on outcomes, including the impact on CR documentation practices of IM residents.

### Limitations

While the models developed have high performance, they are not perfect. However, the intent is for use in formative and not high-stakes summative assessment which would have a higher threshold for implementation [48]. In addition, similar to our earlier work the ML model excludes the interpretive summary which is a component of the original human rubric Revised-IDEA tool but was still considered too complex to tackle with the AI technologies used in this study [5]. Another limitation is that the LLMs used in this study only give a prediction of D and EA scores without explainability. We will experiment further with newer GAI models in next phases of the work that can help address many of these limitations given GAI’s ability to provide narrative explanations and not just classification scores and promising research thus far on the use of GAI in CR assessment with curated data [26,27]. Finally, while we were able to navigate strategies to implement these technologies at 2 sites, there are still some potential limitations that might impact generalizability: (1) not all the technologies we used are readily publicly available and (2) each institution potentially has its own unique writing style which could impact performance of the models and approaches to truncation methods.

### Conclusions

This is the first multi-institutional study to apply LLMs for assessing CR documentation in the EHR. Lessons learned from this study can help promote implementation of these technologies across institutions with ranges of technical resources. Further use of LLMs in the EHR for assessment and feedback can be transformative for medical education and patient care.

## Appendix

Multimedia Appendix 1 The differential diagnosis (D) score from the original Revised-IDEA rubric, a validated tool to assess clinical reasoning documentation, and new explanation of reasoning score (integrating the E score and A score from the original Revised-IDEA rubric).

Multimedia Appendix 2 Additional methods on note preprocessing and truncation.

Multimedia Appendix 3 Additional methods on type NER (named entity recognition) evaluation metric.

Multimedia Appendix 4 Additional methods on model hyperparameters and model packages.

Multimedia Appendix 5 Additional results on selecting thresholds.

## Abbreviations

AI: artificial intelligence
AUPRC: area under the precision-recall curve
AUROC: area under the receiver operating characteristic curve
BERT: bidirectional encoder representation with transformer
BHIDS: Department of Biostatistics, Health Informatics, and Data Sciences
CR: clinical reasoning
DC: diagnostic category
Dx: diagnosis
EHR: electronic health record
GAI: generative artificial intelligence
HIPAA: Health Insurance Portability and Accountability Act
ICC: intraclass correlation
IM: internal medicine
Link: linkage terms
LLM: large language model
ML: machine learning
NER: named entity recognition
NLP: natural language processing
NYU: New York University Grossman School of Medicine
Prior: prioritization of diagnosis language
UC: University of Cincinnati College of Medicine
UCMC: University of Cincinnati Medical Center

## Data Availability

This study involves identified clinical information protected under Health Insurance Portability and Accountability Act regulations and is not available for distribution. Please email the authors with any inquiries about the data or models.
