# Supplementary material for: Large Language Model–Based Assessment of Clinical Reasoning Documentation in the Electronic Health Record Across Two Institutions: Development and Validation Study
Source: J Med Internet Res. 2025 Mar 21;27:e67967. doi: 10.2196/67967 (PMC11971582; doi:10.2196/67967)
Supplement: Multimedia Appendix 1 [file jmir_v27i1e67967_app1.docx]

**Differential Diagnosis:**

Offers more than one relevant diagnostic possibility, committing to what is most likely and considering what is less likely or unlikely yet important to consider for the main chief complaint.

0: No differential

1: Differential is implicitly stated, given as a diagnostic category (e.g. “cardiac”), OR implicitly

prioritized

2: Differential is explicitly stated AND explicitly prioritized

**Explanation of Reasoning:**

Explains the reasoning behind the differential diagnosis, including supporting epidemiology and key features. There is clear linkage of supporting data to the diagnoses in the differential.

0: No explanation of reasoning

1: Explanation of reasoning includes at least 1 data point clearly linked to at least one

diagnosis/diagnostic category on the differential

2: Explanation of reasoning includes at least 1 data point clearly linked to each of at least two of the diagnoses/diagnostic categories on the differential

**Reference:**

1. Schaye V, Miller L, Kudlowitz D, et al. Development of a Clinical Reasoning Documentation Assessment Tool for Resident and Fellow Admission Notes: a Shared Mental Model for Feedback. *J Gen Intern Med.* 2022;37(3):507-512.
