# Supplementary material for: Large Language Model–Based Assessment of Clinical Reasoning Documentation in the Electronic Health Record Across Two Institutions: Development and Validation Study
Source: J Med Internet Res. 2025 Mar 21;27:e67967. doi: 10.2196/67967 (PMC11971582; doi:10.2196/67967)
Supplement: Multimedia Appendix 2 [file jmir_v27i1e67967_app2.docx]

The truncation strategy implemented at NYU was beginning with a start keyword and ending 321 tokens after the start keyword. A list of start keywords such as "presents with", "found to have", or "transferred" (phrases commonly used at the beginning of the assessment and plan) were compiled by human reviewers.^1^ The termination after 321 tokens represented the median count of tokens in the truncated sections following the start keyword in our retrospective dataset.

At UC, truncation used the same start keyword approach with one additional start keyword “medical problems being addressed.” Similarly to NYU, optimal token length following the start keyword was determined through expert annotation of notes to identify the portion showing reasoning of the primary presenting problem. However, we found that the expert annotated optimal token length varied significantly between notes at UC and tended to be relatively short. We found that a ratio of the original length serves as a more accurate estimate of the optimal length, leading us to adopt a ratio-based approach. The truncation strategy implemented, which involved truncating down to 35% of the token length after the start keyword, winsorized between 86 and 283 tokens, the first quartile, and the maximum optimal length, showed a promising improvement. The mean absolute error was reduced to 67.60, a significant improvement compared to the fixed length of 321, which yielded 191.85.

**Reference:**

1. Schaye V, Guzman B, Burk-Rafel J, et al. Development and Validation of a Machine Learning Model for Automated Assessment of Resident Clinical Reasoning Documentation. J Gen Intern Med. 2022;37(9):2230-2238.
