# Supplementary material for: Large Language Model–Based Assessment of Clinical Reasoning Documentation in the Electronic Health Record Across Two Institutions: Development and Validation Study
Source: J Med Internet Res. 2025 Mar 21;27:e67967. doi: 10.2196/67967 (PMC11971582; doi:10.2196/67967)
Supplement: Multimedia Appendix 3 [file jmir_v27i1e67967_app3.docx]

The evaluation of system errors can be broken down into five distinct metrics, each representing a different type of error:

1. Correct (COR): This occurs when the system's output and the golden annotation are identical. For instance, if the system identifies "Aspirin" as a drug and the golden annotation also identifies "Aspirin" as a drug, it is considered Correct (COR).
2. Incorrect (INC): This signifies a discrepancy between the system's output and the golden annotation. For example, if the system identifies "Aspirin" as a brand, but the golden annotation identifies "Aspirin" as a drug, it is considered Incorrect (INC).
3. Partial (PAR): This shows that the system's output and the golden annotation have some similarities, but they are not identical. For instance, if the system identifies "Aspirin tablet" as a drug and the golden annotation identifies "Aspirin" as a drug, it is considered Partial (PAR).
4. Missing (MIS): This means that the system failed to capture a golden annotation. For example, if the system fails to identify "Aspirin" as a drug when the golden annotation does, it is considered Missing (MIS).
5. Spurious (SPU): This occurs when the system generates a response that is not present in the golden annotation. For instance, if the system identifies "Paracetamol" as a drug when the golden annotation does not, it is considered Spurious (SPU).

The Type evaluation schema, in particular, is calculated by determining the degree of overlap between the system-tagged entity and the golden annotation. For instance, if the system identifies "Aspirin tablet" as a drug and the golden annotation identifies "Aspirin" as a drug, the Type evaluation schema would consider this a match because there is some overlap between the system-tagged entity and the golden annotation.

To calculate precision, recall, and F1-score for each evaluation schema, two additional quantities need to be calculated:

1. Possible (POS) = COR + INC + PAR + MIS = True Positive (TP) + False Negative (FN)
2. Actual (ACT) = COR + INC + PAR + SPU = True Positive (TP) + False Positive (FP)

Precision is the ratio of correctly identified named-entities by the Named Entity Recognition (NER) system, while recall is the ratio of named-entities in the golden annotations that the NER system correctly retrieved.

1. Precision = (COR + .5 × PAR) / ACT = TP / (TP + FP)
2. Recall = (COR + .5 × PAR)/POS = COR / ACT = TP / (TP + FN)

For example, if the system identifies 10 entities correctly (COR=10), makes 5 incorrect identifications (INC=5), partially identifies 3 entities (PAR=3), misses 2 entities (MIS=2), and identifies 4 spurious entities (SPU=4), the Possible (POS) and Actual (ACT) quantities would be calculated as follows:

POS = COR + INC + PAR + MIS = 10 + 5 + 3 + 2 = 20
ACT = COR + INC + PAR + SPU = 10 + 5 + 3 + 4 = 22

Then, the precision and recall for exact and partial matches would be calculated as follows:

Precision = (COR + .5 × PAR) / ACT = (10 + .5 * 3) / 22 = .48
Recall = (COR + .5 × PAR)/POS = (10 + .5 * 3) / 20 = .52
