# Supplementary material for: Large Language Model–Based Assessment of Clinical Reasoning Documentation in the Electronic Health Record Across Two Institutions: Development and Validation Study
Source: J Med Internet Res. 2025 Mar 21;27:e67967. doi: 10.2196/67967 (PMC11971582; doi:10.2196/67967)
Supplement: Multimedia Appendix 4 [file jmir_v27i1e67967_app4.docx]

At NYU, each LLM was fine-tuned using the following hyperparameters: a training epoch number of 50, a training batch size of 64, a learning rate of 0.0001, weight decay of 0.01, and a dropout of 0.1. Python 3.8.5 was used for all of our data extraction, model development and validation. Several open-source libraries were utilized: spaCy 3.4.1, scispaCy 0.5.1, scikit-learn 1.0.2, nltk 3.6.5, pandas 1.4.2, HuggingFaceTransformers 4.25.1, torch 1.10.0, and tensorflow 2.5.3.

At UC, the hyperparameters were as follows: a training epoch number of 50, a training batch size of 16, a learning rate of 0.000005 for the encoder layers, 0.0005 for the classifier layer, a weight decay of 0.05, and a dropout of 0.1. Additionally, learning rates were adjusted using an exponential warm-up of 10 epochs and exponential decrease with a 0.9 gamma afterward. Python 3.8.10, scikit-learn 1.3.2, nltk 3.8.1, pandas 1.5.1, HuggingFaceTransformers 4.34.1, torch 2.1.0 were used.
