# Supplementary material for: Large Language Model–Based Assessment of Clinical Reasoning Documentation in the Electronic Health Record Across Two Institutions: Development and Validation Study
Source: J Med Internet Res. 2025 Mar 21;27:e67967. doi: 10.2196/67967 (PMC11971582; doi:10.2196/67967)
Supplement: Multimedia Appendix 5 [file jmir_v27i1e67967_app5.docx]

When selecting the optimal thresholds for all LLM models, both Positive Predictive Value (PPV) and Negative Predictive Value (NPV) that were greater than 69% and False Positives (FP) that are approximately equal to False Negatives (FN) were the major considerations. Such an approach to find the optimal threshold yields balanced errors, which could potentially enhance resident acceptability without compromising the model's performance. An additional advantage of this threshold is its calibration, as the positive and negative rates would closely mirror the actual prevalence of D and EA scores. This implies that the scores aggregated at the trainee level across multiple measures would likely reflect the ground truth accurately. On the NYUTron models the following probability cutoffs were selected for new, unseen data: 0.39 for D0 model, 0.23 for D2 model, and 0.54 for EA2 model, and on the UC EA2 GatorTron model 0.89.
